# Characterizing Inflammatory Cell Asthma Associated Phenotypes in Dental Health Workers Using Cytokine Profiling

**DOI:** 10.3389/falgy.2021.747591

**Published:** 2021-11-18

**Authors:** Tanusha Singh, Braimoh Bello, Mohamed F. Jeebhay

**Affiliations:** ^1^Immunology & Microbiology, National Institute for Occupational Health, National Health Laboratory Service, Johannesburg, South Africa; ^2^Department of Environmental Health, Faculty of Health Sciences, University of Johannesburg, Johannesburg, South Africa; ^3^Department of Clinical Microbiology and Infectious Diseases, School of Pathology, University of the Witwatersrand, Johannesburg, South Africa; ^4^Immunology & Microbiology Department, Centre for Statistical Analysis and Research, Johannesburg, South Africa; ^5^Division of Occupational Medicine and Centre for Environmental & Occupational Health Research, School of Public Health and Family Medicine, University of Cape Town, Cape Town, South Africa

**Keywords:** neutrophilic asthma, eosinophilic asthma, cytokines, work-related asthma, phenotypes, endotypes

## Abstract

Cytokines elicit a pro-inflammatory response by modifying the airway microenvironment in patients with acute or chronic asthma. The expression pattern of several distinct cytokines could be a useful discriminator in asthma. This study aimed to identify asthma subject groupings based on common inflammatory patterns and to determine the relationship between these identified patterns and asthma-associated clinical indices. A sub-group of 76 dental healthcare workers (HCWs) identified from a larger cross-sectional study of 454 dental HCWs in five dental institutions were evaluated further. A self-administered questionnaire elicited the health and employment history of subjects. Sera were analyzed for atopic status, latex sensitization, and 12 cytokines (IL-1β, 3, 4, 5, 6, 7, 8, 10, 12p70, eotaxin, GM-CSF, TNF-α). Pre and post-bronchodilator spirometry was performed on all HCWs. Data clustering and factor analysis were used to identify inflammatory cluster patterns of cytokines. Associations between the cytokine cluster groupings and relevant asthma-associated clinical indices were determined using multivariate logistic regression. The classification of asthma subtype based on cytokine patterns demonstrated both eosinophilic and neutrophilic inflammatory responses. Four phenotypically distinct subgroups relating to the severity of inflammation (acute or chronic) of the cell types were identified. Cytokine determinants for the neutrophilic subtype included IL-1β, 6, 8, 10, 12p70, and TNF-α whereas for the eosinophilic subtype these included IL-3, 4, 5, 7, eotaxin, and GM-CSF. The multivariate models showed a significant association between work-related chest symptoms and all four inflammatory patterns. However, stronger associations were observed for the acute neutrophilic (OR = 6.00, *p* < 0.05) compared to acute and chronic eosinophilic responses (OR = 4.30, *p* < 0.05; OR = 4.93, *p* < 0.05), respectively. Subjects with airway obstruction were more likely to have a mixed cellular infiltrate. The odds of work-exacerbated asthma were increased in acute or chronic eosinophilia (OR = 7.75 and 8.12; *p* < 0.05), respectively as well as with acute neutrophilia (OR = 6) sub-type. This study demonstrated that neutrophilic inflammatory cell asthma phenotypes coexist with eosinophilic inflammatory phenotypes suggesting a possible dual pathway for asthma in dental health workers, probably due to mixed exposures to high molecular weight (e.g., latex) and low molecular weight (e.g., acrylates) agents.

## Introduction

Cytokines play an important role in the inflammatory response of asthmatic airways since they are capable of inducing many pro-inflammatory effects characteristic of the disease (1). These molecules can modify the airway microenvironment directly or by activating resident cells (2). The implementation of tailored medicine requires the identification of endotype-specific markers in biological matrices. In this regard, allergy and asthmatic conditions are well-suited, as they present clinically with similar visible properties (phenotypes) and partially share immune mechanisms (endotypes) (3), thus providing a better understanding of risk prediction, personalized or potentially more effective treatment selection or mechanism-specific prevention strategies (4, 5). Although two major groups of cytokines have been identified based on the pathogenesis of asthma (3), these cytokines cannot be easily categorized in acute and chronic asthma due to their pleiotropic nature and overlapping properties (6). Moreover, cytokine expression patterns consisting of several distinct cytokines are more useful discriminators in multifactorial diseases like asthma compared to single cytokine levels (3, 7).

Asthma has historically been regarded as entirely an atopic disease involving allergic (IgE-mediated) sensitization resulting in interleukin (IL)-5 mediated eosinophilic airways inflammation, which was thought to be a cardinal feature (8). However, the role of IL-5 in asthma has been under scrutiny as studies have demonstrated that only 50% of asthma cases can be attributable to “allergic asthma” (9). This attribution is based on inconsistent associations between different asthma phenotypes possibly due to exposure to high molecular weight (HMW) and low molecular weight (LMW) agents (10). In addition, treatment regimens aimed at neutralizing immunoglobulin E (IgE) (anti-IgE) have only been partially effective in asthma cases. The phenomenon of non-eosinophilic inflammation (referred to as neutrophilic inflammation) is prevalent in certain occupational populations (9). These populations include those with exposure to metal-working fluids (11), LMW sensitizers usually chemical agents (12) such as isocyanates and cobalt (13), acrylates in the dentistry (10, 14, 15) and endotoxin exposure in farming (16). Although not demonstrated in working populations, bakery flour dust has also been shown to elicit a neutrophilic inflammation in mouse models (17). Asthma phenotypes can therefore be classified based on clinical or physiological categories (i.e., severity, age at onset, and chronic airflow obstruction), asthma triggers (i.e., exercise, allergens, occupational allergens or irritants) or partially sharing immune mechanisms (endotypes) of predominant inflammatory cell infiltrates (i.e., eosinophilic or neutrophilic asthma) (18). The latter pathobiological phenotypes include eosinophilic asthma, neutrophilic asthma, granulocytic asthma with concomitant eosinophilic, and neutrophilic inflammation, and a group, paucigranulocytic asthma, in which levels of both neutrophils and eosinophils are normal (9, 19, 20).

The measurement of sputum eosinophilic cationic protein (ECP) has been suggested as a predictive marker for the presence of eosinophilic inflammation in the lung (21) and changes in sputum eosinophilia also reflect fluctuations in asthma control (3). Furthermore, ECP is more predictive of sputum eosinophilia than serum IgE (3). Hancox et al., demonstrated that high blood eosinophil counts were associated with airflow obstruction (lower forced expiratory volume in one second [FEV_1_] % predicted values for both pre- and post-bronchodialator spirometry, *p* ≤ 0.048) (22). The pathobiological effects of eosinophils occur through a myriad of mediators including granulocyte-macrophage colony-stimulating factor (GM-CSF), IL-3, IL-5, and certain chemokines such as eotaxin (3, 6, 23, 24). Neutrophils also play a role in the development of well-defined clinical sub-phenotypes (25–30) and are a potential source of inflammatory mediators, enzymes (e.g., neutrophil elastase, myeloperoxidase [MPO]), reactive oxygen species, and cytokines such as IL-8 and tumor necrosis factor alpha (TNF-α). Interleukin-8 is considered a marker of severe asthma and has been linked to allergic rhinitis (27, 31).

The use of biomarkers in the assessment of airway inflammation is important for exploring the underlying mechanisms of asthma and may reveal diseased airways that are not detectable by parameters commonly used in current practice (e.g., symptom experience, lung function, airway hyperresponsiveness) (32). Airway-infiltrating cells like eosinophils modify airway responses and have profound biologic effects in the micro-environment of their release as well as at distant sites. However, they may no longer be present at the time that the effects of the cytokine/s are apparent, which has probably contributed to discordance in results reported in previous studies. The discordance may also be attributed to the heterogeneity of asthma (7, 19, 33), highlighting the need to make inferences about the relationship between airway hyperresponsiveness and airway inflammation to be made with circumspection (34). A potential advantage of using inflammatory cell markers is the ability to distinguish between heterogeneous subtypes in subjects with similar clinical characteristics (7). This study hypothesized that different cytokine groupings could be used to recognize different inflammatory cell endotypes in asthmatics and these groupings could be used to examine the heterogeneity of airway inflammation in dental healthcare workers (HCWs). The aim of the study was to identify asthma subject groupings based on common inflammatory patterns and to determine the relationship between these identified patterns and asthma-associated clinical indices.

## Materials and Methods

### Study Population

This study was part of a larger cross-sectional study of 454 employed dental HCWs (34). Subjects were assessed using a standardized protocol that included an interviewer-administered questionnaire, pulmonary function tests, immunological assessment for atopy and sensitization to occupational allergens (latex, chloramines). Further detailed information as to the methods employed in identifying individuals with asthma are described in this communication. In brief, the questionnaire collected a history of previous medical illnesses and other symptom variables: respiratory (wheeze and/or tight chest); ocular (itchy eyes, red eyes); and nasal (runny nose, blocked nose/stuffy nose) symptoms. The current study population comprised 76 asthmatic subjects, that were identified using epidemiological definitions of asthma as either having doctor-diagnosed asthma, probable asthma or those with current asthma-related symptoms (35). Probable asthma was defined as having reported either an attack of shortness of breath, attack of asthma or using asthma medication. Those with current asthma-related symptoms included those with a positive response to one or more of the following: shortness of breath, chest tightness, coughing, and wheezing in the last 12 months. Health outcome information was obtained from a self-administered respiratory health questionnaire based on the European Community Respiratory Health Survey (ECRHS) and spirometry indices obtained according to the American Thoracic Society (ATS) guidelines (36) as described previously (34). A positive bronchodilator test entailed an increase in FEV_1_ and/or forced vital capacity (FVC) ≥12% and ≥200 ml increase from baseline and is described below. Control subjects (*n* = 9) were classified as those with a negative response to either doctor-diagnosed asthma, probable asthma or current asthma-related symptoms.

### Serum Samples

A 6 ml blood sample was taken from all workers using a Becton Dickinson Vacutainer CAT tube (with clot activator). Samples were left to coagulate for 3–6 h at room temperature and were then centrifuged at 1,500 g for 10 min. The serum was transferred to 5 ml plastic cryotubes (Nunc, Denmark) and stored at −70°C until analysis.

#### Measurement of Atopy and Allergen-Specific IgE

The serum was analyzed for atopy and probable latex allergy using the Phadiatop® test for common inhalants and latex-specific IgE ImmunoCAP (k82), respectively. Tests were done according to the manufacturer's instructions using the ImmunoCAP system 100 (Phadia AB, Uppsala, Sweden). Specific IgE to cross-reacting carbohydrate determinants (CCDs) and horse-radish peroxidase (HRP) and bromelain (MUXF^3^) were also performed. A result of >0.35 kU/l was regarded as positive for allergen-specific IgE determinations.

#### Measurement of Eosinophilic Cationic Protein (ECP)

Serum ECP was measured using the Phadia ImmunoCAP system ECP FEIA (Phadia AB, Uppsala, Sweden), according to the manufacturer's instructions. Briefly, anti-ECP covalently coupled to ImmunoCAP, binds with the ECP in the patient's serum. After washing, enzyme-labeled antibodies against ECP were added to form a complex. After incubation, unbound enzyme-anti-ECP was washed away and the bound complex was then incubated with a developing solution. The reaction was stopped, the fluorescence of the eluate was measured and the response transformed to concentration levels using a calibration curve. The normal range for serum ECP in the patient population that was established in the laboratory was 2.00–15.7 μg/l. Therefore, values above this were considered elevated for the study population. The maximum value was similar to that of a previous study (13.3 μg/l) (37). To ensure quality control the laboratory participated in an external quality assessment program (Phadia: Quality Club ECP [52-5213-99/05]).

#### Measurement of Myeloperoxidase (MPO)

MPO concentration in serum was measured using the MPO enzyme immunoassay (MPO-EIA) (MPO-EIA, Oxford Biomedical Research), following the manufacturer's instructions (Oxford Biomedical Research). Briefly, antigen captured by a solid phase monoclonal antibody was detected with a biotin-labeled goat polyclonal anti-MPO. An avidin alkaline phosphatase conjugate was bound to the biotinylated antibody. The alkaline phosphatase substrate ρ-nitrophenyl phosphate (pNPP) together with the yellow product (ρ-nitrophenol) was read at 405 nm using a spectrophotometer (Biotek Instruments, United States of America). A standard curve was obtained by plotting the absorbance values as a function of the standard MPO concentrations. Standard curves were fitted using a four-parameter data reduction method. Assays in which the standard curve had a correlation coefficient (*R*^2^) ≥ 0.98 and samples with a coefficient of variation (CV) ≤ 10% were accepted. The normal range for serum in the patient population that was established in the laboratory was 8.43–77.35 ng/ml and values above this were considered elevated for the study population.

#### Measurement of Non-allergic and Allergic Inflammatory Mediators (Cytokines)

The BD^TM^ CBA Human Inflammation kit was used to quantitatively measure non-allergic inflammatory cytokines (interleukin (IL)-8, IL-1β, IL-6, IL-10, TNF-α, and IL-12p70) in each serum sample. The assay allowed for multiplexed analysis of multiple proteins from a single sample. Six bead populations with distinct fluorescence intensities coated with the specific ILs were multiplexed and resolved in the red channel of the flow cytometer (BD FacsArray^TM^) (Becton Dickson, Oxford, United Kingdom). The determination of the serum inflammatory ILs was done according to the kit's instructions. Standard curves of the standard serial dilutions, expressed by a four-parameter logistic model (log CC = D + (A – D)/(1 + (log I/C)^∧^B)), where A = minimum asymptote, B = slope factor, C = inflection point, D = maximum asymptote, were used to determine the limit of detection (LOD) for each specific analyte based on the average median fluorescent intensity (MFI) of the negative control. The assay detection limit for each analyte was determined by the average fitted concentration of the negative control (0 pg/mL) + 2 standard deviations for each analyte. The concentration of analyte was expressed as picograms per milliliter (pg/mL). A regression coefficient (*R*^2^) for the standard curve for each analyte was accepted if ≥0.98. The results were interpreted by the level of expression of the cytokine with categories of low expression to highly expressed cytokines (0–2.9, >2.9–4.5, >4.5–6.5, >6.5–9.4, >9.4–13.2, >13.2–28, >28–80.4, >80.4–429 pg/ml). The allergy-related inflammatory cytokines (IL-7, IL-3, IL-4, IL-5, eotaxin, and GM-CSF) were measured using the BD^TM^ CBA Human Soluble Protein Flex Set assay. The multiplexed bead populations were resolved in the near-infrared (NIR) and red channels of the flow cytometer (BD FacsArray^TM^) (Becton Dickson, Oxford, UK). The procedure was done according to the kit's instructions. The results were interpreted as described for the non-allergic cytokine mediators above.

### Spirometry and Bronchodilator Challenge (BDR)

Spirometry was conducted according to the American Thoracic Society (ATS) guidelines using a Jaeger Masterscope spirometer (Höchberg, Germany), calibrated at least twice a day with a 3-L syringe. Reproducibility criteria included the two best tracings for both FEV_1_ and FVC varying by no more than 0.2 L (38). Measurements obtained by spirometry were adjusted according to the temperature and atmospheric pressure measured throughout the day. Special instructions were given to workers to ensure that they did not smoke tobacco (at least 1 h) and did not use any anti-asthmatic inhalers (4 h) or take oral asthma medications (8 h) before the test. Workers' heights were recorded for calculating predicted lung function indices using reference values of the European Community for Coal and Steel with lower limits corresponding to the 95^th^ percentile (39). The post-bronchodilator test was done at least 10 min after administration of the bronchodilator (inhaled beta-2 agonists, salbutamol, 400 mg). An improvement of at least 12% and 200 ml after administration of salbutamol was regarded as a significant response. Both pre- and post bronchodilator FEV_1_ had to be reproducible for calculating the degree of the bronchodilator response.

### Statistical Analysis

Variables were log-transformed to meet the model assumptions of normality and parametric analyses were then applied. However, where certain variables followed a non-guassian distribution after transformation, non-parametric analyses were used. The results of the continuous variables were expressed as means and standard deviations. Chi-squared statistics were applied for binary comparisons. The grouping of subjects based on their cytokine profiles was achieved by both agglomerative hierarchical complete linkage clustering (40) and partitional clustering to compare how well the algorithms performed on the cytokine measurements. For the former technique, the identification was based on the largest stopping rule index (Calinski/Harabasz pseudo-F = 118.67). The study sample was also divided into four clusters using partition clustering (kmedians), based on previously known inflammatory cell asthma phenotypes (neutrophilic, eosinophilic, granulocytic and paucigranulocytic) (18). Factor analysis was used to determine inflammatory patterns based on the 12 cytokines (41, 42). Principal factor analysis (PFA), as well as principal component factor analysis (PCA), was done to determine the inflammatory cell phenotypes. The appropriateness of the correlation matrix (0.015) for factor analysis was assessed by the Bartlett's test of sphericity (*p* < 0.001) and the Kaiser-Meyer-Olkin test (0.75). The number of factors was determined by the Eigenvalue criterion, whose magnitude should be ≥1, as well as the percentage of total variance explained by the factors. To increase the interpretability, the factors were rotated orthogonally through the Varimax rotation procedure. The following criteria were used in labeling the factors: i) a factor loading had to be >0.45; ii) variables that loaded >0.45 on more than one factor were excluded, and iii) single factor loadings were not accepted (43). The naming of factors was based on the general association between the various cytokines and severity of asthma cited in the literature (44–46). To test for the difference between cluster groupings by phenotypic characteristics, analysis of variance (ANOVA) (with Bonferroni correction) was used. This method is similar to bi-clustering where not only the objects are clustered but also the features of the objects (i.e., the rows and columns are clustered simultaneously) (47). To further determine the association between the cluster groupings and cytokine patterns with asthma-associated clinical indices, multivariate logistic regression analysis, adjusting for age and gender, was applied. Statistical analysis was performed using STATA9 computer software (StataCorp, 2007, Texas, USA).

## Results

### Demographic Characteristics

The age distribution between the asthmatic subjects and controls was similar (Table 1). There was a larger proportion of females than males in both groups. The asthmatic workers had a marginally higher average body mass index (BMI), placing them in the overweight category, compared to the control group that was borderline normal. The majority of subjects from both groups were non-smokers. The control group had relatively better lung function compared to the asthmatic group, although this was not statistically significant. Just over half of the asthmatic subjects were atopic (59%) and ~ a tenth (9%) were sensitized to latex. The two cross-reacting carbohydrate determinants (CCDs), horse-radish peroxidase (HRP) and bromelain (MUXF^3^) were measured in all latex sensitized asthmatic subjects to exclude cross-reactivity. Furthermore, both serum inflammatory biomarkers (ECP, MPO) were relatively higher in the asthmatic group whereas the cytokine levels were variable between the two groups.

**Table 1:** Demographic characteristics of dental healthcare workers.

**Demographic**	**Asthma subjects**	**Controls**
**characteristics**	***n* = 76**	***n* = 9**
**Clinical characteristics**		
Age (years)	34 ± 13.00	33 ± 12.67
**Sex**		
- Male	26 (34.2%)	1 (11.1%)
- Female	50 (65.8%)	8 (88.9%)
Body mass index	26 ± 6.13	24 ± 4.23
**Smoking history**		
- Current	12 (15.8%)	1 (11.1%)
- Ex-smoker	4 (5.3%)	-
- Non-smoker	55 (72.4%)	7 (77.8%)
**Allergy**		
Latex	7 (9.2%)	N/A
Atopy	44 (57.8%)	N/A
**Cross-reacting carbohydrate determinants (CCDs)**		
MUXF	7 (9.2%)	-
HRP	7 (9.2%)	-
**Upper respiratory symptoms**		
- Ocular-nasal symptoms	32 (42.1%)	-
- Work-related ocular-nasal symptoms	7 (9.2%)	-
**Lower respiratory symptoms**		
- Asthma-related symptoms	28 (36.8%)	-
- Work-related chest symptoms	12 (15.8%)	-
**Lung function**		
- FEV_1_ predicted (%)	86.18 ± 17.21	91.53 ± 14.49
- FVC predicted (%)	90.77 ± 14.21	93.13 ± 9.23
- FEV_1_/FVC (%)	98.88 ± 13.85	104.99 ± 11.38
**Serum inflammatory biomarkers**		
- ECP (kU/L)	10.57 ± 13.40	4.20 ± 4.50
- MPO (μg/L)	48.96 ± 38.27	29.76 ± 23.24
**Cytokines**		
- IL-1β	4.92 ± 9.69	3.57 ± 5.89
- IL-3	3.32 ± 7.37	4.03 ± 5.16
- IL-4	3.08 ± 3.91	4.39 ± 3.96
- IL-5	1.99 ± 3.39	2.10 ± 2.15
- IL-6	5.57 ± 9.51	4.23 ± 4.17
- IL-7	11.77 ± 9.92	12.20 ± 8.61
- IL-8	12.94 ± 21.86	4.41 ± 5.50
- IL-10	3.93 ± 3.12	5.47 ± 3.20
- IL-12p70	9.31 ± 5.90	11.21 ± 6.64
- TNF-α	7.46 ± 5.91	9.86 ± 5.41
- Eotaxin	22.53 ± 34.75	38.57 ± 35.19
- GM-CSF	9.22 ± 49.17	10.94 ± 23.58

*Continuous variables, mean and standard deviation; Categorical variables, number (%); HRP, horseradish peroxide; MUXF, bromelain; N/A, not applicable; Body mass index, Underweight (<18.5), normal (18.5–24.9), overweight (25.0–29.9), obese (>30.0); Work-related ocular-nasal symptoms (itchy eyes, red eyes, runny nose, blocked nose / stuffy nose); Work-related chest symptoms (tight chest or wheezing when at work or job change due to work-related symptoms or improvement after changing jobs). FEV_1_, forced expiratory volume in 1s; FVC, forced vital capacity; Reference values of the ECCS (FEV_1_ / FVC < 0.7 and FEV_1_ < 80% predicted)*.

### Cytokine Groupings Defined by Cluster Analysis

**Figure 1:**
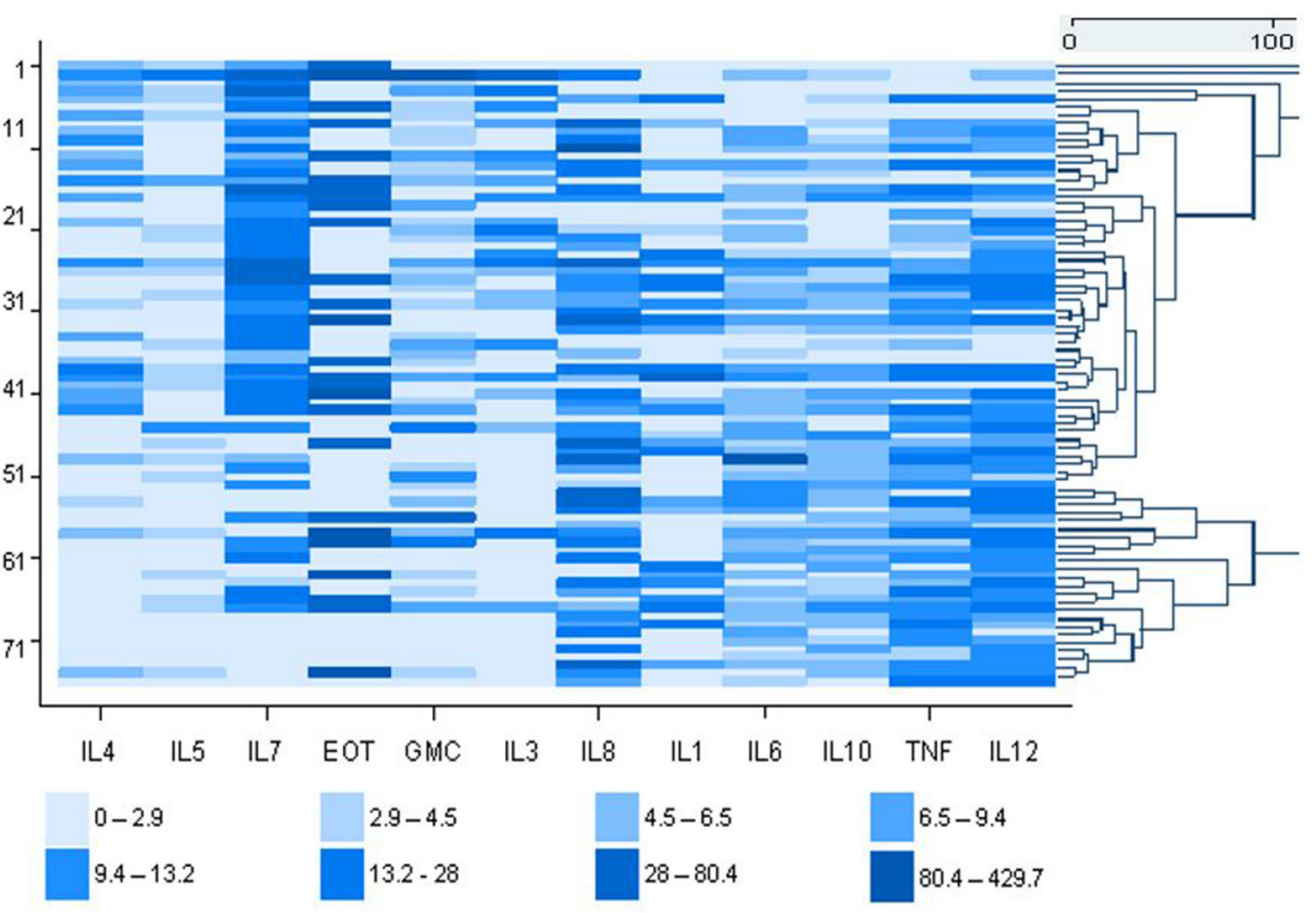
Heat map showing the molecular profile of 12 inflammatory markers concentrations in asthmatic dental workers (cases). Each value is represented by a colored rectangular block. The high expressing cytokines (pg/ml) are depicted by the darker color shades and the low expressing cytokines by the lighter shades. Each row is an individual dental worker and the individual cytokine values are shown in each column. To lhe right of the heatmap, is the dendrogram showing the distinction between groups.

The expressions of 12 cytokines are shown in the heat map, which illustrates the variability of the measured cytokines in the asthmatic study population (*n* = 76) (Figure 1). The agglomerative clustering analysis procedure identified four potential clusters within the population. However, the agglomerative clustering produced background noise making it difficult to distinguish between groups. The population was therefore partitioned into four discernable groups using partition clustering namely cluster-1, cluster-2, cluster-3, and cluster-4. This provided some optimality criterion and therefore the clusters identified by this method were used for further analysis in this investigation.

### Identification of Inflammatory Patterns

**Figure 2:**
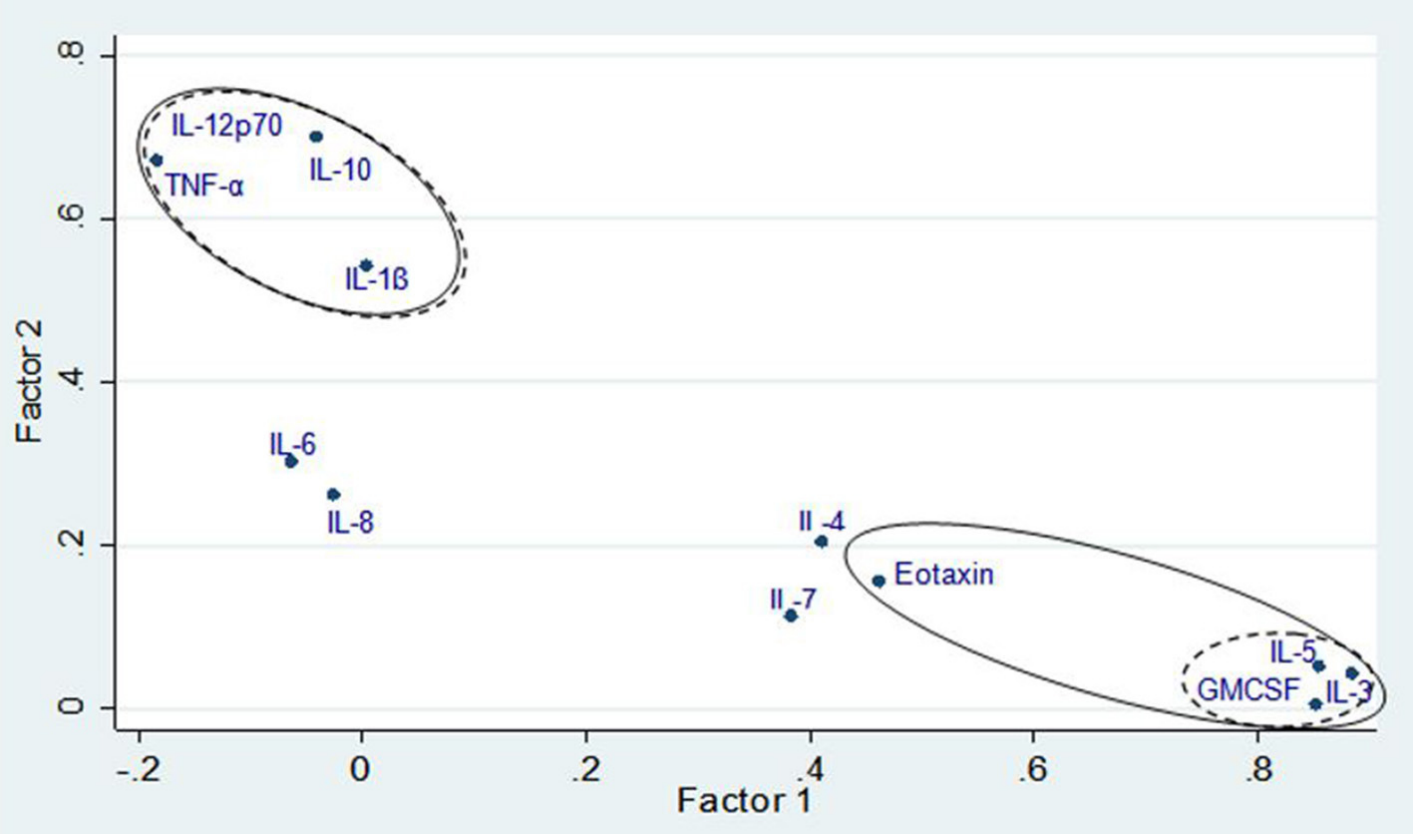
Factor loading plot for principal factor analysis showing factor groupings with and without Varimax rotation. Solid line denotes variable loadings included in factors without rotations and dashed lines denote variable loadings included in factors with Varimax rotation.

The correlation matrix of cytokines was first subjected to PFA for the 76 subjects (Figure 2). A 2-factor model was confirmed based on the Eigen values for factor-1 (F1) and factor-2 (F2) with 2.83 and 1.95, respectively. The following cytokines met the factor selection (correlation (ρ) > 0.45), F1: IL-5, eotaxin, GM-CSF, IL-3 and F2: IL-1β, IL-10, TNF-α, and IL-12p70. The exception was eotaxin which featured in F1 but did not meet the selection criteria when Varimax rotation was applied (ρ = 0.43). Further analysis was done to extract the highest possible variance in the dataset using PCA and Varimax rotation. A four-factor (F1, F2, F3, and F4) solution was found to represent the data based on Eigenvalue criteria, explaining ~ 67% of the variance in the data set (*p* < 0.001). The Varimax rotation demonstrated unique factor loadings in F3 for IL-4 and IL-7, which were excluded from the initial PFA (Table 2). The labeling of the four factors identified by Varimax rotation was based on the factor selection criteria and the relation of the inflammatory patterns in asthma (Table 2).

**Table 2:** Principal component factor loading with Varimax rotation and factor labeling according to the inflammatory response of the cytokine groupings in dental healthcare workers.

**Variable**	**F1: chronic**	**F2: acute**	**F3: acute**	**F4: chronic**
	**eosinophilia**	**neutrophilia**	**eosinophilia**	**neutrophilia**
IL-5	**0.89**	−0.04	0.15	0.04
Eotaxin	**0.55**	0.23	0.11	−0.34
GMCSF	**0.93**	−0.07	0.01	0.03
IL-3	**0.88**	−0.05	0.22	−0.03
IL-4	0.29	0.17	**0.67**	−0.14
IL-7	0.21	−0.04	**0.81**	0.13
IL-8	−0.01	0.13	0.16	**0.76**
IL-6	0.06	0.26	−0.15	**0.63**
IL-1β	−0.03	**0.71**	0.22	−0.29
IL-10	0.01	**0.75**	0.11	0.22
TNF-α	−0.14	**0.75**	0.04	0.21
IL-12p70	−0.03	**0.80**	−0.19	0.12

*The bold values represent the four factors with Eigenvalue criterion ≥1 using Varimax rotation*.

### Relationship Between Cluster Groupings and Inflammatory Patterns

To determine whether there were differences between the four inflammatory patterns and the four cluster groupings an ANOVA test was conducted (Table 3). All the cytokines that scored high in F1 (chronic eosinophilia) demonstrated the highest mean levels in subjects from cluster-1. Interleukin-6 and IL-8, which were highly expressed in F4 (chronic neutrophilia) showed the highest mean levels for subjects in cluster-3. There was a significant variance in the cytokine measurements featured in F2 and F4 and the cluster groupings. The majority of cytokines that scored high in F2 (acute neutrophilia) were highly expressed in cluster-4. These results demonstrated that there are distinct differences between the clusters and the majority of the factors.

**Table 3:** Analysis of variance between cytokine measurements of the identified factors, by the four cluster groupings in dental healthcare workers.

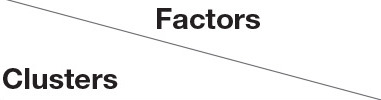	**Cluster-1**	**Cluster-2**	**Cluster-3**	**Cluster-4**	***p*-value**
	***n* = 26**	***n* = 31**	***n* = 2**	***n* = 17**	
**F1:** chronic eosinophilia					
IL-5	**3.1 ± 4.70**	1.7 ± 2.61	2.6 ± 0.58	0.7 ± 1.61	0.139
Eotaxin	**65.9 ± 25.57**	0	0	0	<0.001
GM-CSF	**21.6 ± 83.52**	3.9 ± 4.97	1.3 ± 1.85	1.0 ± 2.32	0.474
IL-3	**5.9 ± 10.76**	2.5 ± 4.76	0	1.2 ± 3.60	0.149
**F2:** acute neutrophilia					
IL-1β	7.7 ± 14.50	1.4 ± 3.81	0	**7.6 ± 6.11**	0.044
IL-10	4.1 ± 3.45	3.0 ± 2.56	5.4 ± 0.78	**5.3 ± 3.24**	0.061
TNF-α	7.3 ± 6.73	5.9 ± 4.32	**12.2 ± 1.77**	10.0 ± 6.61	0.076
IL-12p70	9.2 ± 6.74	7.6 ± 5.03	9.4 ± 4.03	**12.6 ± 5.16**	0.004
**F3:** acute eosinophilia					
IL-4	**4.5 ± 3.85**	2.2 ± 3.40	2.7 ± 3.77	2.5 ± 4.52	0.468
IL-7	13.6 ± 9.29	**14.2 ± 9.43**	14.3 ± 11.85	4.4 ± 8.74	0.002
**F4:** chronic neutrophilia					
IL-8	10.6 ± 15.17	5.9 ± 5.45	**103.5 ± 88.59**	18.7 ± 13.01	<0.001
IL-6	3.7 ± 2.98	4.3 ± 2.65	**43.9 ± 55.50**	6.1 ± 3.45	<0.003

*IL-7, IL-10 and TNF-α were not normally distributed after transformation hence non-parametric tests were used. Arithmetic means and standard deviations are displayed*.

*The bold value represent cytokines which score in the factor grouping and demonstrated the highest mean levels in the respective clusters, demonstrating distinct differences between clusters and the majority of the factors*.

### Relationship Between Serum Inflammatory Markers and Individual Cytokines

The relationship between serum inflammatory markers (ECP and MPO) and individual cytokines determined are presented in Table 4. A significant positive correlation was demonstrated between IL-8 and both ECP (*r* = 0.42, *p* < 0.001) and MPO (*r* = 0.47, *p* < 0.05). Similarly, a modest correlation was found between allergic inflammatory cytokines (IL-5, IL-7) and MPO. An inverse correlation was demonstrated between IL-1β and MPO (*r* = −0.40, *p* < 0.05).

**Table 4:** Correlation between log transformed serum inflammatory markers (ECP and MPO) and cytokine concentrations for the various factors identified in dental healthcare workers.

**Factors**	**Pearson correlation**	
	**coefficient (** ***r*** **) (** ***n*** **)**	
	**ECP (kU/l)**	**MPO (ng/ml)**
**F1**: chronic eosinophilia		
IL-5	−0.01 (40)	0.30 (42)^*^
Eotaxin	0.03 (30)	0.29 (32)
GM-CSF	−0.02 (50)	0.17 (52)
IL-3	0.01 (24)	0.15 (26)
**F2**: acute neutrophilia		
IL-1β	−0.25 (30)	–0.40 (31)^*^
IL-10	−0.08 (59)	0.08 (60)
TNF-α	−0.13 (62)	−0.24 (63)
IL-12p70	−0.19 (70)	−0.12 (71)
**F3**: acute eosinophilia		
IL-4	0.18 (42)	0.11 (43)
IL-7	0.12 (62)	0.25 (62)^*^
**F4**: chronic neutrophilia		
IL-8	0.42 (57)^**^	0.47 (58)^*^
IL-6	0.16 (66)	0.13 (67)

* *p < 0.05*,

** *p < 0.001*.

### Relationship Between Inflammatory Patterns and Asthma-Associated Clinical Indices

Examination of the asthma-associated clinical indices and cytokine inflammatory patterns revealed a significant association between having work-related chest symptoms and all four inflammatory patterns (Table 5). Subjects with airway obstruction were significantly (*p* < 0.05) more likely to have a mixed cellular infiltrate of acute and chronic eosinophilia and acute neutrophilia. The odds of having increased MPO were higher in subjects with either acute and chronic neutrophilic inflammation, although this was not significant. The frequency of asthma attacks appeared to be more common in subjects with acute or chronic eosinophilia (OR range: 7.42–9.49) in the unadjusted model (data not shown). The odds of having atopic asthma were 10-fold higher in subjects with a chronic eosinophilic response and appeared to be similar in both acute eosinophilic and neutrophilic responses (OR = ~5). Furthermore, the odds of WEA were similar in subjects with acute or chronic eosinophilia (OR = 7.75 and 8.12; *p* < 0.05), respectively as well as in acute neutrophilia (OR = 6). Similar observations were demonstrated in other unadjusted logistic models (data not shown).

**Table 5:** Association between asthma related clinical indices and inflammatory phenotypes identified in dental healthcare workers (*n* = 76) in multivariate logistic regression models.

**Health outcomes**	**Inflammatory phenotype (OR, CI)**			
	**F1 *Chronic eosinophilia* OR (95% CI)**	**F2 *Acute neutrophilia* OR (95% CI)**	**F3 *Acute eosinophilia* OR (95% CI)**	**F4 *Chronic neutrophilia* OR (95% CI)**
**Upper respiratory symptoms**				
- Ocular-nasal symptoms	1.42 (0.60–3.34)	1.67 (0.60–4.61)	2.13 (0.98–4.63)	2.15 (0.13–34.76)
- Work-related ocular-nasal symptoms	0.27 (0.04–2.04)	0.77 (0.17–3.51)	0.72 (0.21–2.49)	5.23 (0.32–85.01)
**Lower respiratory symptoms**				
- Asthma-related symptoms	1.92 (0.82–4.50)	0.70 (0.22–2.26)	1.69 (0.76–3.74)	ND
- Work-related chest symptoms	4.93 (1.45–16.77)^*^	6.00 (1.51–23.80)^*^	4.30 (1.32–14.01)^*^	ND^*^
- Number of asthma attacks	1.64 (0.23–11.96)	ND	1.32 (0.17–10.26)	ND
**Lung function**				
- FEV_1_/FVC <0.7	8.61 (1.45–51.06)^*^	3.19 (0.35–29.11)	5.79 (1.43–23.52)^*^	ND^*^
- FEV_1_ <80% predicted	0.95 (0.34–2.64)	0.67 (0.15–3.04)	1.52 (0.62–3.72)	4.62 (0.28–75.21)
**Atopy**	1.76 (0.74–4.18)	1.26 (0.43–3.69)	1.49 (0.67–3.31)	1.39 (0.08–23.19)
**Latex sensitization**				
- Composite latex (k82) extract	0.76 (0.17–3.47)	0.54 (0.67–4.33)	1.47 (0.47–4.57)	ND
- True latex sensitization	ND	ND	1.45 (0.12–17.90)	ND
**Serum inflammatory biomarkers**				
- ECP	1.38 (0.52–3.68)	0.99 (0.27–3.62)	1.15 (0.45–2.97)	5.14 (0.31–84.49)
- MPO	1.56 (0.55–4.46)	2.35 (0.71–7.76)	0.48 (0.11–2.12)	8.35 (0.50–138.85)
**Asthma phenotypes**				
- Atopic asthma	11.93 (4.44–32.05)^**^	5.94 (1.49–23.76)^*^	4.39 (1.48–13.07)^*^	ND
- Non-atopic asthma	ND	1.39 (0.17–11.30)	2.64 (0.72–9.69)	ND^*^
- Work-exacerbated asthma	8.12 (2.19–30.09)^**^	6.00 (1.16–31.09)^*^	7.75 (2.19–27.45)^*^	ND

*Each OR is derived from a separate model adjusted for age and gender*;

* *p < 0.05*;

** *p < 0.001. ND, OR not determinable; Asthma-related symptoms, yes to any of: “Have you had an attack of asthma in the last 12 months?”; “Have you been woken by an attack of shortness of breath in the last 12 months?”; “Have you been woken with a feeling of tightness in your chest at any time in the last 12 months?” or “Have you been woken by an attack of coughing at any time in the last 12 months?”*.

*Frequency of asthma attack in past 12 months defined as “frequent” if more than one attack per month and “none” if no attack per month*.

*ECP and MPO categorical variable: positive response based on values above the normal range of the patient population*.

## Discussion

This study demonstrated four cluster groupings based on differing cytokine patterns in dental health workers with asthma-associated clinical indices, resulting in multiple inflammatory phenotypes. The asthma subtypes based on cytokine measurements entailed two related to an eosinophilic and the other two to a neutrophilic response. Each cellular response also appeared to be related further to the degree of inflammation (i.e., acute or chronic) present. Similar results have been shown by Brasier et al., in which at least four phenotypically distinct subgroups were identified based on bronchoalveolar lavage-derived cytokines for severe asthma (6). Shared variance among clusters and cross-loadings of factors also suggested that some cytokines may overlap between groups or represent aetiological pathways that have smaller effects (48–50). Lemiere et al., identified three clusters in occupational asthmatics based on increases in fractional exhaled nitric oxide (FeNO) following challenge to causal agents, two clusters showing an increase post challenge to high molecular weight (HMW) agents, the two differing in degrees of severity of response, and a third cluster presenting with late reactions due to LMW agents (50). Vandenplas et al., also demonstrated differences in work-related asthma symptom patterns since subjects with OA due to LMW agents were more likely to experience chest tightness at work and daily sputum production (10). The results of the current study confirm the utility of the factor analysis (43) approach in identifying the various inflammatory patterns of asthma in dental HCWs.

Soluble markers such as ECP and MPO are generally used to assess the presence of eosinophils and neutrophils in body fluids, respectively (32, 51, 52). In the current study, a significant correlation was observed between cytokines generally associated with chronic neutrophilia with both ECP and MPO levels (53). Furthermore, a significant correlation was found between acute as well as chronic eosinophilic cytokines and MPO. A combination of cellular types (54) generally reflects an inflammatory response to multiple agents characterized by dual inflammatory pathways as a result of mixed exposures. The dual response could either indicate independent pathways caused by concomitant exposure to both HMW and LMW agents in this setting or synergistic effects between exposures that may be augmenting the inflammatory response. The primary HMW agent in this context is considered to be bacterial endotoxin (9), which has been reported previously to act as an adjuvant for certain allergens (55). An exposure characterization study conducted by our group also confirmed higher endotoxin exposure levels in dental health workers (56). It has also been observed that neutrophils can also be a source of ECP in IgE-mediated responses (57), possibly due to HMW and LMW concomitant exposures. This suggests that the nature of airway inflammation observed in dental health workers may be due to mixed (HMW and LMW) exposures. Furthermore, Suojalehto et al., have recently shown that phenotypic characteristics of acrylate-induced occupational asthma, when compared to other LMW agents, were more similar to occupational asthma caused by HMW agents (15). The role that multiple workplace exposures plays in the manifestation of different inflammatory types of asthma is further elaborated by other investigators (58, 59). The differences between acrylate exposure and other LMW agents common in dental health care settings, has therefore challenged the historical practice of pooling exposures into a single group, on the assumption that they all share similar pathophysiologic mechanisms (15).

In this study, a strong correlation was observed between acute eosinophilic inflammation and asthma-associated clinical indices for work-relatedness including work-related chest symptoms (acute eosinophilia: OR = 4.30, chronic eosinophilia: OR = 4.93), airway obstruction (acute eosinophilia: OR = 5.79, chronic eosinophilia: OR = 8.61), atopic asthma and work-exacerbated asthma (WEA) (acute eosinophilia: OR = 7.75, chronic eosinophilia: OR = 8.12). These clinical indices suggest a dominant allergic component due to its eosinophilic nature caused by agents such as common inhalants, latex and acrylates among others (60). These results are also consistent with finding from the data reported by Hancox et al., that higher eosinophils are associated with lower FEV_1_ values (22). Interestingly, ECP and MPO did not demonstrate a significant association with any of the asthma-associated clinical indices, except MPO which was positively associated with non-atopic asthma (OR = 3.06, 95% CI: 1.119–8.363, *p* < 0.05) (data not shown). This lack of association suggests that these two serum biomarkers do not appear to be useful as predictive markers as they may not be present when the effects of these cytokines manifest (7, 19, 33). It would also suggest that cytokine production, is mainly responsible for bronchial hyperresponsiveness (29, 61).

Cytokines commonly associated with eosinophilic inflammation in asthma include IL-3, IL-5, eotaxin, and GM-CSF (6, 23, 24). These cytokines were particularly prominent in the factor named chronic eosinophilic inflammation. Furthermore, subjects with chronic eosinophilia also had an increased odds of having atopic asthma. The other group of cytokines associated with acute eosinophilic inflammation was IL-4 and IL-7. These cytokines have been reported to synergize the induction of IgE and IgG4 production in a T-cell-dependent manner (62). Overexpression of IL-4 in the lungs is associated with a lymphocytic and eosinophilic inflammation, without necessarily causing airway hyperreactivity (23, 63). Hancox et al., have demonstrated that eosinophilia is a risk factor for airflow obstruction even among those without symptoms (22). Furthermore, IL-4 is generally associated with atopic diseases (e.g., allergic rhinitis and atopic dermatitis) (64). This was also observed in the current study, in that the odds of having atopic asthma were increased in both acute and chronic eosinophilic groups relative to the other groups. A previous study has shown that bronchoalveolar lavage samples with elevated CD4+ cells that expressed both IL-4 and IL-17 predicted greater asthma severity (65). Although not investigated in this current study, IL-17 appears to be an independent risk factor for severe asthma and warrants further investigation in occupational populations.

The cytokines associated with the neutrophilic responses in this study included IL-1β, IL-10, TNF-α, IL-12p70 and IL-8, IL-6, which may be potentially caused by agents such as endotoxin and mercury among others (12, 15). The results demonstrated that both acute and chronic neutrophilic groups were more likely to be associated with work-related factors (although less so than the eosinophilic responses) in the absence of HMW agents playing a major role in asthma. Furthermore, subjects with chronic neutrophilic inflammation were more likely to have work-related chest symptoms and non-atopic asthma. Taylor et al., have also shown that neutrophilic asthma is associated with an airway microbiome that is significantly different compared to patients with other inflammatory phenotypes (66). Since endotoxins, a by-product of Gram-negative microorganisms, are likely to result in a neutrophilic response. These findings are therefore consistent with other studies that non-allergic asthma associated with endotoxin exposure, possibly be due to contamination of dental unit waterlines (56, 67), is mediated by an inflammatory response involving several cytokines, including IL-1, IL-6, IL-8, and TNF-α, IL-12 (25, 68).

This study has highlighted the use of biomarkers in subjects with similar clinical characteristics of asthma to ascertain the pathophysiological basis for the symptoms in dental health care settings. While previous studies have used factor analysis to identify distinct asthma phenotypes focusing on clinical measures and quality of life, duration of the asthmatic attack and steroid use (32), this study has further extended the utility of this approach. It has demonstrated the presence of a complex cytokine network and various inflammatory subtypes in asthmatic dental HCWs. This suggests that asthma in this occupational setting is not exclusively due to an eosinophilic inflammatory response. The mixed inflammatory profile may be the result of responses to concomitant exposures to multiple different allergens or may also represent synergistic effects between allergens and agents such as endotoxin. Factors expressed as quantitative traits in the form of cytokine inflammatory patterns to characterize asthma phenotypes can therefore be useful in the exploration of asthma causation in working populations. Furthermore, it is also probable that the level of exposure to multiple agents may also result in the manifestation of a gradation of immune responses. Potential limitations of this study is the cross-sectional nature of the study design and the small study population (especially of the control group), the latter producing some unstable effect estimates that could not be interpreted optimally. Furthermore, the broad definition for asthma may have resulted in disease misclassification, although it could have also contributed to improved sensitivity of identifying subjects with asthma.

In conclusion, this study has demonstrated that dental health workers with similar clinical characteristics of asthma can have heterogeneous subtypes, denoting inflammatory patterns associated with different causative agents, especially when exposed to multiple agents co-exists. Aside from improved diagnosis, this can also contribute toward effective prevention strategies at a population level should the causative agents in this setting be identified.

## Data Availability Statement

The datasets presented in this article are not readily available because of the Protection of Personal Information Act and restrictions apply to the availability of these data, which were used for the ethics application. Data are however available from the authors upon reasonable request and with permission of the University of the Witwatersrand Ethics Committee. Requests to access the datasets should be directed to tanushas@nioh.ac.za.

## Ethics Statement

The studies involving human participants were reviewed and approved by University of the Witswatersrand, Human Research Ethics Committee, Medical (Ref: R14/49), University of Kwazulu-Natal, University of the Western Cape, University of Pretoria (Ref: 32/2005), University of Michigan Medical School Institutional Review Board (H04-00006341-I). The dental institutions granted permission to conduct the study and the patients/participants provided their written informed consent to participate in this study.

## Author Contributions

All authors listed have made a substantial, direct and intellectual contribution to the work, and approved it for publication.

## Funding

This project was supported by an NIH Research Grant #2 D43 TW00812-06 through the Fogarty International Center ITREOH Programme awarded to the University of Michigan. Other sources of funding include the Allergy Society of South Africa-Glaxo Smith Kline Research Award and the National Institute for Occupational Health, NHLS.

## Conflict of Interest

The authors declare that the research was conducted in the absence of any commercial or financial relationships that could be construed as a potential conflict of interest.

## Publisher's Note

All claims expressed in this article are solely those of the authors and do not necessarily represent those of their affiliated organizations, or those of the publisher, the editors and the reviewers. Any product that may be evaluated in this article, or claim that may be made by its manufacturer, is not guaranteed or endorsed by the publisher.
